# Ultrasonic evaluation of the abductor hallucis muscle in hallux valgus: a cross-sectional observational study

**DOI:** 10.1186/1471-2474-14-45

**Published:** 2013-01-28

**Authors:** Sarah Stewart, Richard Ellis, Mike Heath, Keith Rome

**Affiliations:** 1 School of Podiatry, Health & Rehabilitation Research Institute, AUT University, Auckland, New Zealand; 2 School of Physiotherapy, Health & Rehabilitation Research Institute, AUT University, Auckland, New Zealand; 3 Horizon Scanning, Health & Rehabilitation Research Institute, AUT University, Auckland, New Zealand

**Keywords:** Hallux valgus, Abductor hallucis, Musculoskeletal ultrasound, Intrinsic foot muscle, First metatarsophalangeal joint

## Abstract

**Background:**

The aim of the study was to investigate the abductor hallucis muscle characteristics, defined as dorso-plantar (DP) thickness, medio-lateral (ML) width, and cross-sectional area (CSA) in relation to the severity of hallux valgus using musculoskeletal ultrasound. One hundred and two feet, mean (SD) age of 60.3 (20.54) years old, displaying varying severities of hallux valgus were stratified into four groups representing the four grades of the Manchester Scale (grade 0: no deformity, grade 1: mild deformity, grade 2: moderate deformity and grade 3: severe deformity).

**Methods:**

The abductor hallucis muscle was imaged in each foot using a portable ultrasound system. The mean (SD) DP thickness, ML width, and CSA measurements were compared across the four Manchester Scale grades using a one-way ANOVA.

**Results:**

Significant differences in DP thickness were found between feet with no hallux valgus (grade 0) and feet with hallux valgus grade 2 (p = 0.001) and 3 (p < 0.001). Significant differences were also found in ML width between feet with no hallux valgus (grade 0) and feet with grade 2 hallux valgus (p = 0.010). Significant differences in CSA were found between feet with no hallux valgus (grade 0) and feet with grade 2 (p < 0.001) and grade 3 (p < 0.001) hallux valgus. No significant differences in these three muscle characteristics were found between grades 1, 2 and 3 (p > 0.0125).

**Conclusions:**

We speculate that morphological changes to the abductor hallucis muscle occur early in the development of the deformity.

## Background

Hallux valgus is a forefoot deformity involving progressive lateral deviation of the hallux and medial deviation of the first metatarsal resulting in a medial prominence at the first metatarsophalangeal joint (1MTPJ) [1]. The deformity is more common in the older population with estimated prevalence being 36% in those over 65 years, while the prevalence rate in adults between 18 and 65 years is 23% [2]. The presence of juvenile hallux valgus has been estimated to occur in 8% of those under 18 years [2]. Although the deformity may remain asymptomatic, it has been associated with cosmetic appearance concerns, pain, and reduced health-related quality of life [3-5]. Hallux valgus may also contribute to postural instability and increase the risk of falls in the older population [6].

Several risk factors have been identified in the aetiology of hallux valgus including increasing age [2], being female [7], a positive family history of hallux valgus [8], abnormal hindfoot kinematics [9], wearing high-heeled shoes [10,11] or tight footwear [12,13]. Bony abnormalities (round-shaped first metatarsal head [14], long first metatarsal [15], bipartite medial sesamoids [16]) and muscular abnormalities (accessory extensor hallucis longus tendon [17], accessory tibialis posterior tendon [18]) have also been linked to the deformity. It is likely that a combination of factors contribute to the development of hallux valgus.

The abductor hallucis muscle, which originates from the medial calcaneal tuberosity and inserts onto the medial sesamoid and/or the proximal phalanx [19], plays an important role through isometric contraction in maintaining 1MTPJ stability and preventing abnormal transverse plane motion [20]. With progressive valgus deviation of the hallux the abductor hallucis muscle loses its normal anatomical relationship with the 1MTPJ, shifting to the plantar aspect of the first metatarsal rather than medial to it. The muscle therefore loses its abductory force and instead gains flexor force [20]. Despite the involvement of the abductor hallucis muscle in the pathomechanics of the deformity, a limited number of studies have investigated the abductor hallucis muscle with hallux valgus [19-24].

Previous studies have employed a variety of techniques to investigate the abductor hallucis muscle including electromyography (EMG) [20,22,25-28], magnetic resonance imaging (MRI) [29], and musculoskeletal ultrasound [23,24,30,31]. Previous research has shown good correlation of soft tissue imaging as well as similar reliability between MRI and ultrasound [32,33]. Studies have also shown that ultrasound has similar validity to EMG and manual muscle testing in assessing neuromuscular pathologies in extrinsic and intrinsic foot muscles with an added advantage in its ability to visualise muscle atrophy [34,35]. Therefore, the purpose of this study is to employ musculoskeletal ultrasound to determine significant differences between dorso-plantar (DP) thickness, medio-lateral (ML) width and cross-sectional area (CSA) of the abductor hallucis muscle between different severities of hallux valgus. We hypothesise that there will be a significant difference in DP thickness, ML width, and CSA of the abductor hallucis muscle between different severities of hallux valgus.

## Methods

### Participants

64 participants (52 females and 12 males) aged over 20 years old, were recruited from the Auckland University of Technology Podiatry Clinic. Selective sampling was utilised to recruit participants with varying severities of hallux valgus deformity. All participants provided written informed consent, and ethical approval was obtained from the Auckland University of Technology Ethics Committee (AUTEC12/111). Participants were excluded with a history of foot or ankle surgery; current trauma to the foot and ankle; a neuromuscular condition, or a diagnosis of inflammatory arthritis, diabetes mellitus.

### Methodology

Hallux valgus was measured using the Manchester Scale which utilises four photographs of increasing severity of hallux valgus [36]. This tool has been shown to have excellent reliability [30] and validity [14,37] in both clinical assessment and self-assessment of hallux valgus [38]. The scale is graded 0 ‘no deformity’, 1 ‘mild deformity’, 2 ‘moderate deformity’, and 3 ‘severe deformity’ [36]. The researcher (SS) observed each participant in relaxed weight-bearing stance to determine which of one of the four Manchester Scale photographs best represented the degree of hallux valgus deformity [36]. A Chison 8300 Deluxe Digital Portable Ultrasound System (Jiang Su, China) with a 50 mm linear probe of 7.5 MHz was used to obtain images of the abductor hallucis muscle belly. The researcher underwent 3 months of training in performing ultrasound scans prior to data collection. The ultrasound machine has been shown to produce reliable images of the abductor hallucis muscle for the purpose of measuring ML width, DP thickness, and CSA [24]. A similar procedure to that outlined by Cameron [23] was used by the researcher (SS) to obtain ultrasound images in the current study. This involved the participant being instructed to fully relax in a seated position with the legs extended. The foot to be measured was positioned with the ankle at neutral (i.e. 0°). The knees were supported in approximately 15° of flexion with the involved leg in a comfortable degree of external rotation to optimise the researcher’s access to the medial foot during scanning. The researcher palpated the medial malleolus and using a ruler drew a line anterior to this bony landmark in an inferior direction. Parker Aquasonic® 100 Ultrasound Transmission Gel (Fairfield, USA) was applied along this drawn line to optimise skin-probe contact whilst avoiding compression of the muscle. The probe was positioned perpendicular to the drawn line. Three repetitive images were obtained for each foot and a mean value calculated. A 30 second rest was allowed between each image capture in which the probe was placed back in its holder.

**Figure 1:**
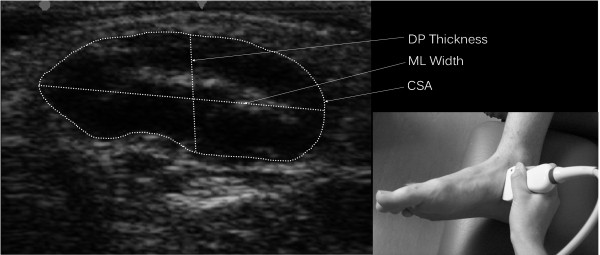
Muscle characteristic measurements with inset showing probe placement.

Image J v. 1.45 (National Institutes of Health, Bethesda, MD, USA), an image processing and analysis software, was used to measure the ML width, DP thickness, and CSA measurements of the abductor hallucis muscle from the ultrasound images captured on the machine. To ensure researcher blinding all images were randomised before analysis. Each image was analysed to obtain measurements for DP thickness (mm), ML width (mm), and CSA (mm^2^) (Figure 1). The edges of the muscle were defined as the point between the muscle tissue and the muscle fascia. The DP thickness was determined using a line selection tool to connect the dorsal most aspect of the muscle with the plantar most aspect of the muscle. The ML width was calculated in the same manner using the tool to connect the medial most aspect of the muscle with the lateral most aspect of the muscle. CSA was measured manually using an area selection tool to trace around the muscle border.

### Statistical analysis

All data analysis was conducted using the Statistical Package for Social Sciences (v.20, SPSS Inc., Chicago, IL, USA). To determine the intra-rater reliability for measurements of ML width, DP thickness, and CSA of the abductor hallucis muscle, intraclass correlation coefficients (ICC_3,1_) were used. In addition, standard error of measurement (SEM) calculations were undertaken to provide a direct representation of measurement error. A one-way ANOVA was conducted to compare the differences in the mean values of DP thickness, ML width and CSA between the four hallux valgus grades using the 5% level of significance. A Bonferroni post hoc correction was used to determine the differences between the four grades of hallux valgus with significance at p < 0.0125.

## Results

**Figure 2:**
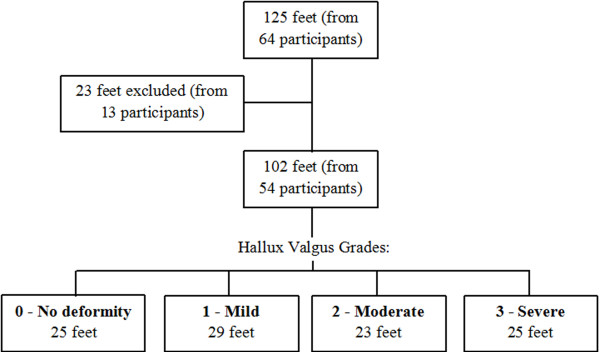
Flow of participants through the study.

Of the ultrasound images obtained from 125 feet, images from 23 feet were excluded as they displayed high echo intensity making it difficult to visualise the muscle borders for measurement analysis. Figure 2 displays the flow of participants through the study. Table 1 displays the demographic and clinical data of the 102 included feet. Abductor hallucis muscle measurements showed high intra-tester reliability for DP thickness (ICC_3,1_ 0.94, SEM 0.45 mm), ML width (ICC_3,1_ 0.92, SEM 0.90 mm), and CSA (ICC_3,1_ 0.96, SEM 12.33 mm^2^). All data demonstrated normal distribution. Descriptive statistics for the abductor hallucis muscle characteristic measurements are displayed in Table 2. A significant difference was found between the four hallux valgus grades for mean DP thickness (F = 7.474, df = 3, p < 0.001), mean ML width (F = 4.560, df = 3, p = 0.005) and mean CSA (F = 7.862, df = 3, p < 0.001). Post hoc analysis revealed a significant difference in DP thickness between feet with grade 0 and grade 2 (p = 0.001) and grade 3 (p < 0.001). For ML width a significant difference was found between grade 0 and grade 1 (p = 0.010), while for CSA significant differences were found between grade 0 and grade 1 (p < 0.001) and grade 0 and grade 3 (p < 0.001).

**Table 1:** Demographic characteristics

**Demographic Characteristics**	**Value**
Age (years), Mean (SD)	60.3 (20.5)
Gender (F: M), (%)	(83: 19), (81% women)
Ethnicity, n (%)	European, 91 (89%)
	Asian, 10 (10%)
	Māori, 1 (1%)
BMI (Kg/mm^2^), Mean (SD)	26.33 (5.27)

**Table 2:** Descriptive statistics for the abductor hallucis muscle characteristics

	**Grade 0, Mean (SD)**	**Grade 1, Mean (SD)**	**Grade 2, Mean (SD)**	**Grade 3, Mean (SD)**
*n*	25	29	23	25
DP thickness (mm)	13.3 (2.0)	11.9 (1.4)	11.4 (1.6)	11.3 (1.7)
ML thickness (mm)	33.2 (2.3)	32.2 (2.5)	30.4 (4.0)	30.8 (3.3)
CSA (mm^2^)	339.3 (56.4)	300.1 (46.1)	271.5 (61.4)	272.9 (61.5)

## Discussion

Significant differences were found in DP thickness, ML width, and CSA of the abductor hallucis muscle between feet without hallux valgus compared to feet with hallux valgus. We propose that these results may be attributed to the muscle’s changing anatomical position in feet with hallux valgus which reduces its capacity to be recruited during abduction of the hallux [20]. As muscle size is considered an important determinant of muscle strength, these findings may suggest that hallux valgus is associated with a reduction in strength of the abductor hallucis muscle as a result of muscle disuse. A decline in muscle function secondary to disuse is similar to that associated with increasing age [39].

Although significant differences were found in DP thickness, and CSA of the muscle between those without deformity and those with moderate and severe deformity, differences in ML width were found only between those without hallux valgus and those with moderate deformity. It may be that the width of the muscle is not as accurate a represenation of muscle size as thickness and CSA measurements, or it may be that the width of the muscle is not affected by muscle disuse to the same extent as the thickness or area.

As the muscle size characteristics of the abductor hallucis muscle did not vary significantly between the mild, moderate and severe stages of hallux valgus it may be that morphological changes to the muscle occur early in the development of the deformity and do not change significantly thereafter. We therefore propose that intervention strategies which aim to improve the strength and function of the muscle be implemented in the mild stage of the deformity (i.e. grade 1). Although previous studies have demonstrated foot exercises can activate the abductor hallucis muscle, these studies were conducted on participants with pes planus (flatfeet) [27] or asymptomatic feet [28]. Heo [28] reported that the highest electromyographic activation of the muscle occurred with isolated abduction of the hallux against resistance. However, voluntary abduction of the hallux, which is deemed to be challenging in healthy individuals [20], may also prove difficult to those with foot deformity. The effectiveness of this exercise in recruiting and strengthening the muscle in those with hallux valgus is likely to be affected by the changing anatomical position of the abductor hallucis muscle. Jung [30] demonstrated that eight weeks of abductor hallucis muscle strengthening with the use of short foot exercises and/or foot orthotics increased the CSA of the abductor hallucis muscle in feet with pes planus. Although short foot exercises, which involve a flexion component at the first 1MTPJ, may recruit the abductor hallucis muscle to a greater extent than abduction exercises in feet with hallux valgus, further research is warranted to determine whether short foot exercises would have the same effect on abductor hallucis CSA in those with foot deformity.

A feasibility study assessing ultrasound of lower leg and foot muscles reported that images obtained of the abductor hallucis muscle from participants over the age of 60 years displayed increased echo intensity and 18% were excluded from measurement analysis due to poor visualisation of the muscle borders [31]. We obtained similar findings. Verhulst [31] also reported that age was the most determinant factor for echo intensity. This can be explained by increased infiltration of fat and collagen which accompanies age-related loss of skeletal muscle mass [40]. It is unclear whether increased adipose deposition in the abductor hallucis muscle is associated with hallux valgus, or age-related changes. However, it has been noted that muscle adipose deposition is associated with a decline in muscle strength and function in older adults suggesting such changes may not be favourable to those with hallux valgus. [39,41] We attempted to recruit participants of a wide range of ages in each of the four hallux valgus groups, however the mean age of participants was not equal across the groups with mean age rising as the stage of deformity increased. Although this is consistent with the identification of increasing age as a risk factor for hallux valgus, it is possible that increasing age may have contributed to the decreasing muscle size characteristics. Lower limb muscle mass has been found to decrease by 25% from age 20 to 70 years [39].

The results of this study should be considered in light of limitations. The cause and effect relationship between abductor hallucis muscle size reduction and hallux valgus severity could not be assessed due to the cross-sectional nature of the study design. Furthermore, from the original images, 18% were excluded from the analysis because they displayed high echo intensity making it difficult to visualise the muscle boundaries for measurement analysis. This is suggestive of increased adipose and collagen tissue within the muscle which creates multiple interfaces and increased reflection of the ultrasound beam [42].

A limitation of 2D imaging was obtaining images that represented the same cross-sectional slice of the abductor hallucis muscle across the participants. Although we used a standardised procedure to image the muscle, the placement of the probe along the reference line varied due to the individual differences in the position of the abductor hallucis muscle in relation to this line. Not only does the muscle move inferiorly in feet with hallux valgus, but individual differences have also been shown in the course of the muscle (whether arciform or straight) [43]. However, this limitation did not appear to greatly influence the reliability of the image measurements in the current study, despite randomisation of the order of the images being analysed, coupled with the researcher being blinded to the feet the images were depicting.

A final limitation was that foot length and width was not measured so it is unknown whether the muscle size characteristics differ between foot size.

Future work may employ the use of electromyography in conjunction with ultrasound to assess functional parameters of the abductor hallucis muscle during various strengthening exercises to determine which exercise best activates the muscle in those with hallux valgus. Future research may also examine ultrasonic size characteristics of other muscles involved in the development of hallux valgus (i.e. hallux flexors and extensors, adductor hallucis) as these muscles also lose their normal anatomical relationship with the 1MTPJ.

## Conclusions

Participants with hallux valgus display significantly reduced ML width, DP thickness and CSA of the abductor hallucis muscle than individuals without hallux valgus. As these muscle characteristics do not significantly differ between mild, moderate, and severe stages of hallux valgus, we speculate that morphological changes to the abductor hallucis muscle may occur early in the development of the deformity. Our findings may be explained by the muscle’s change in position within hallux valgus feet and the resultant loss of abductory function. Therefore, interventions designed to increase abductor hallucis muscle strength early on in the deformity may benefit those with hallux valgus.

## Competing interests

The authors declare that they have no competing interests.

## Authors’ contributions

SS carried out the data collection, performed the statistical analysis and contributed to the interpretation of the data. RE participated in supervision of the research project, collection of ultrasound data, and drafting of the manuscript. MH provided technical training in ultrasound. KR participated in the design of the study, supervision of the research project, and drafting of the manuscript. All authors read and approved the final manuscript.

## Pre-publication history

The pre-publication history for this paper can be accessed here:

http://www.biomedcentral.com/1471-2474/14/45/prepub
